# Silver Nanoparticle-Based Fluorescence-Quenching Lateral Flow Immunoassay for Sensitive Detection of Ochratoxin A in Grape Juice and Wine

**DOI:** 10.3390/toxins9030083

**Published:** 2017-02-28

**Authors:** Hu Jiang, Xiangmin Li, Ying Xiong, Ke Pei, Lijuan Nie, Yonghua Xiong

**Affiliations:** 1 State Key Laboratory of Food Science and Technology, Nanchang University, Nanchang 330047, China; riverhu@163.com (H.J.); lixiangmin73@163.com (X.L.); yingxiong201468@163.com (Y.X.) pk415917ibs@126.com (K.P.); 2 Jiangxi-OAI Joint Research Institute, Nanchang University, Nanchang 330047, China; lijuannie2016@163.com

**Keywords:** silver nanoparticles, fluorescence quenching, lateral flow immunoassay, ochratoxin A, quantitative detection

## Abstract

A silver nanoparticle (AgNP)-based fluorescence-quenching lateral flow immunoassay with competitive format (cLFIA) was developed for sensitive detection of ochratoxin A (OTA) in grape juice and wine samples in the present study. The Ru(phen)${}_{3}^{2+}$-doped silica nanoparticles (RuNPs) were sprayed on the test and control line zones as background fluorescence signals. The AgNPs were designed as the fluorescence quenchers of RuNPs because they can block the exciting light transferring to the RuNP molecules. The proposed method exhibited high sensitivity for OTA detection, with a detection limit of 0.06 µg/L under optimized conditions. The method also exhibited a good linear range for OTA quantitative analysis from 0.08 µg/L to 5.0 µg/L. The reliability of the fluorescence-quenching cLFIA method was evaluated through analysis of the OTA-spiked red grape wine and juice samples. The average recoveries ranged from 88.0% to 110.0% in red grape wine and from 92.0% to 110.0% in grape juice. Meanwhile, less than a 10% coefficient variation indicated an acceptable precision of the cLFIA method. In summary, the new AgNP-based fluorescence-quenching cLFIA is a simple, rapid, sensitive, and accurate method for quantitative detection of OTA in grape juice and wine or other foodstuffs.

## 1. Introduction

The lateral flow immunoassay (LFIA) is a very useful on-site screening method in clinical diagnosis, antiterrorism detection, and environmental and food safety monitoring because it is a low-cost technique for naked-eye detection and it exhibits rapid and simple operation in resource-constrained countries [1,2,3,4,5]. Among LFIAs, LFIAs with competitive format (cLFIA) are commonly used to detect small chemical molecules (hapten), such as veterinary, illegal drugs, antibiotics, and mycotoxins. However, the cLFIA frequently suffers from relatively low sensitivity because it requires a large amount of target analytes to eliminate the signals on the test (T) line (signal “turn off”) by competitively binding the antibody on the probe [6]. Recently, some research groups reported a novel fluorescence-quenching LFIA sensor in improving the sensitivity of the cLFIA method based on the signal “turn-on” mode. The fluorescent materials are either sprayed on the T and control (C) zones or precoated on the entire nitrocellulose (NC) membrane as background signals [7,8,9,10] in this sensor. In theory, a cLFIA with a “turn-on” mode would produce a higher sensitivity compared with that of a “turn-off” mode because the fluorescent signal on the T line with a “turn-on” mode is proportional to the analyte concentration. For example, Fu et al. used a fluorescent nanoparticle as the donor to develop a fluorescence-quenching cLFIA. The limit of detections (LODs) for the heavy metals chromium and clenbuterol were 1.56 and 0.04 µg/L, respectively [7,8]. Zhang et al. used the fluorescein microspheres as the fluorescent donor for β2-adrenergic agonist detection, and the LOD was 0.12 µg/L [10]. Most of the above-mentioned published studies believed that the primary cause of fluorescence quenching is attributed to the fluorescence resonance energy transfer (FRET) between AuNPs and fluorescein or fluorescein-doped microspheres. The distance between the donor and acceptor is an important factor for generating a high quenching efficiency because the FRET commonly occurs in close proximity (within 10 nm) [11]. However, the FRET efficiency using fluorescein coated on an entire nitrocellulose membrane as background fluorescence cannot be high because the size of antibody is about 8~10 nm [12]. If the fluorescent microspheres are used as background fluorescence, almost no FRET can occur because the distance between AuNPs and dyes is large than 10 nm. 

Therefore, we suggest the inner-filter effect is the main cause of fluorescence quenching in the strip assay. The phenomenon of the inner-filter effect (IFE) will occur when the absorption spectrum of metal nanoparticles overlaps with the fluorescence excitation or emission spectra of fluorescent material [13]. To prove this concept, we proposed the use of Ru(phen)${}_{3}^{2+}$-doped silica nanoparticles (RuNPs) as fluorescent donors in the present study. RuNPs as transition metal–based luminescent labels exhibit many advantages, such as a large Stokes’ shift, a high emission quantum yield and a long fluorescence lifetime [14]. The maximum excitation and emission wavelengths of RuNPs are 450 and 590 nm, respectively. For the first time, silver nanoparticles (AgNPs) were used as the extinction nanomaterial to absorb the excitation light of the strip reader. Thereby, the fluorescence emission of RuNPs is blocked because the surface plasmon resonance (SPR) absorption peak of AgNPs totally overlaps with the excitation spectrum of RuNPs. The AgNPs process a higher molar extinction coefficient compared with that of conventional gold nanoparticles [15]. Thus, using AgNPs as fluorescence quenchers can result in a strong extinction ability and high sensitivity. 

Ochratoxin A (OTA) is one of the most common mycotoxins with strong nephrotoxic, teratogenic, and immunosuppressive properties and it has been classified as a possible human carcinogen (group 2B) by the International Agency for Research on Cancer [16]. OTA is also suspected to be involved in Balkan endemic nephropathy and in the high frequency of urinary tract tumors observed in some Balkan areas [17]. OTA can contaminate a variety of crops, including maize, wheat, coffee, and fruits, particularly grapes [18,19,20,21,22,23,24]. Many countries have set maximum admissible levels of OTA to manage its effects. For example, the European Union has established a maximum admissible level of as low as 2 μg/L OTA in grape juice and wine. A novel AgNP-based fluorescence-quenching cLFIA method (AgNP–RuNP–cLFIA) was developed for sensitive detection of OTA in grape juice and wine to maximize the sensitivity of the cLFIA for OTA detection. The 63.6 nm AgNPs were used as the quenchers of the fluorescence of RuNPs. The performance of the AgNP–RuNP–cLFIA sensor was evaluated in terms of specificity, detection limit, linear range, accuracy, and precision for OTA quantitative detection in grape juice and wine samples.

## 2. Results and Discussion

### 2.1. Scheme of AgNP–RuNP–cLFIA Sensor

**Figure 1:**
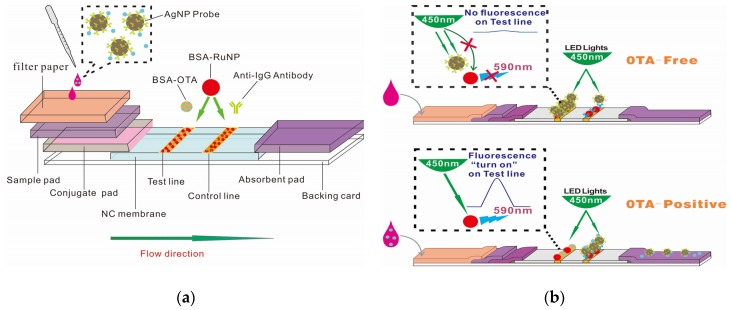
Schematic of the AgNP–RuNP–cLFIA sensor: (**a**) fabrication; (**b**) detection of ochratoxin A (OTA)-free and OTA-positive samples.

The principle of the AgNP–RuNP–cLFIA sensor is shown in Figure 1b. The AgNP probe will migrate on the NC membrane and will be captured by the bovine serum albumin (BSA)–OTA antigen on the T line when an OTA-free sample is applied to the sample well. The fluorescence intensities (FIs) of the T lines are completely quenched by the antibody-coated AgNPs because the AgNPs will absorb the photons from the light-emitting diode (LED) arrays and block the photon transfer to RuNPs, thereby resulting in the absence of a fluorescence signal in the T zone. The OTA molecule in the solution can competitively bind the antibody on the AgNPs and reduce the amount of AgNP probes on the T line when an OTA-positive sample is added into the well. The fluorescent signal will be turned on, and the FI value on the test line is proportional to the OTA concentration.

### 2.2. Characterization of AgNPs, RuNPs, and AgNP Probes

**Figure 2:**
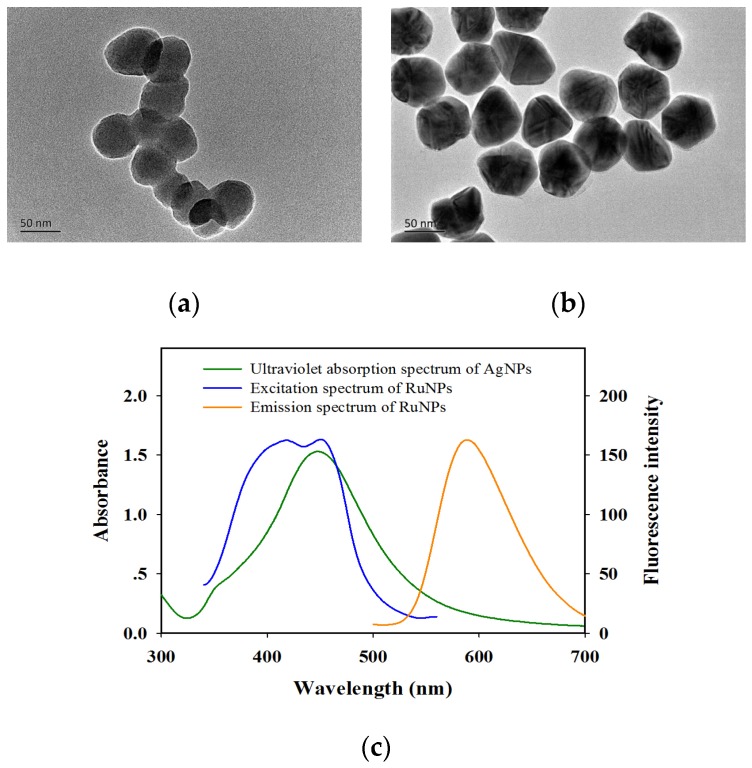
Characterization of Ru(phen)${}_{3}^{2+}$-doped silica nanoparticles (RuNPs) and silver nanoparticles (AgNPs) used in AgNP–RuNP–cLFIA sensor: (**a**) Transmission electron microscopy (TEM) images of RuNPs; (**b**) TEM images of AgNPs; (**c**) Ultraviolet absorption spectrum of the AgNPs and fluorescence excitation and emission spectra of the RuNPs.

The transmission electron microscopy (TEM) image in Figure 2a shows that the average diameter of BSA–RuNPs was 54.4 ± 5.2 nm (*n* = 50). The fluorescence spectrum of BSA–RuNPs in Figure 2c indicates that the maximum excitation and emission wavelengths were 450 and 590 nm, respectively. A commercial strip reader with 450 nm LED arrays was used as an exciting light source to scan the cLFIA sensor to obtain a strong fluorescent signal. The AgNPs were prepared through 11 round growths with the addition of silver seeds, sodium citrate, tannic acid (C_76_H_52_O_46_), and silver nitrate (AgNO_3_) solution. The as-prepared AgNPs were well distributed and exhibited relatively uniform spheres with an average diameter of 63.6 ± 3.7 nm (*n* = 50), as shown in Figure 2b. The UV–vis spectrum demonstrates that the maximum SPR peak of AgNPs is 450 nm (Figure 2c). The SPR absorption peak of AgNP totally overlaps with the RuNP excitation peak. 

**Figure 3:**
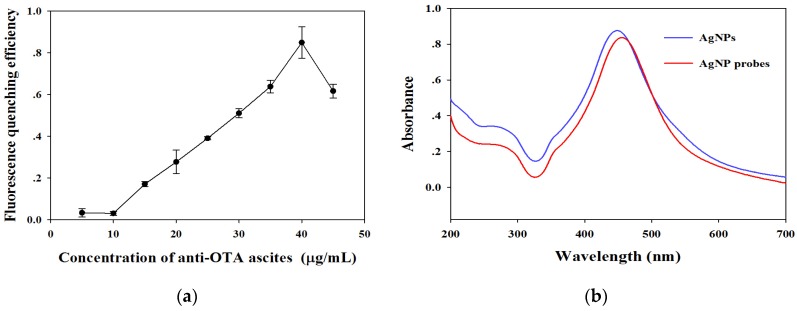
(**a**) Optimization of the amount of anti-OTA ascites labeled on the AgNPs. The vertical bars indicate the standard deviation (*n* = 3); (**b**) Ultraviolet absorption spectra of AgNPs before and after labeling anti-OTA ascites.

The AgNP probes were prepared by labeling the unpurified anti-OTA ascites into the AgNPs directly. The saturated amounts of anti-OTA ascitics labeled into the AgNPs were optimized by dropping 0 μg to 45 μg of anti-OTA ascitics to 1 mL AgNP solution (OD_450_ = 7.5). The optimal amounts of anti-OTA ascitics labeled on AgNPs were evaluated by calculating the fluorescence-quenching efficiency of the resultant AgNP probes to fluorescent signals on the T line. The fluorescence-quenching efficiency on the T line increased from 3.3% to 84.9% with labeled amounts of anti-OTA ascitics increasing from 5 μg to 40 μg as shown in Figure 3a. The fluorescence-quenching efficiency sharply decreased to 61.6% when the labeled ascitic amounts were further increased to 45 μg. This phenomenon indicates that the excessive protein amounts on the surface of AgNPs can produce a steric hindrance and decrease the bioactivity of probes. Thus, 40 μg of ascites per mL of AgNP solution was verified as the optimal labeled ascitic amount and was used for subsequent experiments. The SPR band of AgNP probes showed a 5 nm redshift under the optimal labeling condition (Figure 3b). The redshift band further confirmed that the anti-OTA ascitics were successfully labeled on the surface of AgNPs.

### 2.3. Parameter Optimization of AgNP–RuNP–cLFIA Sensor

The background FIs on both lines, the amounts of AgNP probes, and the BSA–OTA concentration on the T line were considered the most important factors that affected the sensitivity of the cLFIA sensor. First, the amounts of BSA-RuNP on both lines were optimized by spraying a different concentration of BSA-RuNP solution into the NC membrane as the dispensing density of T and C lines was 0.75 μL/cm. The NC membrane was then dried with a vacuum dryer at 37 °C for 2 h. The FI value on the T line increased from 124 to 1786 as the BSA-RuNP concentration increased from 20 μg/mL to 320 μg/mL, as shown in Figure S1. A low fluorescent signal on the T line indicates a low signal-to-noise ratio, which would cause a narrow linear range and a large error. Meanwhile, a strong fluorescence signal could lead to a low sensitivity because of more probe consumption. The fluorescent signals on both lines were approximately 480 when the BSA-RuNP concentration was 80 μg/mL, and two significant red fluorescence bands were observed under 450 nm LED arrays (the stereogram displayed in Supplementary Material Figure S1). Thus, 80 μg/mL of BSA-RuNP solution was selected as the optimal concentration to produce the background fluorescent signals on both lines. A similar “checkerboard titration” was performed with different AgNP probe contents under a series of BSA-OTA concentrations on the T line for various combinations to further achieve the best sensitivity. The FI values of the T line for OTA-free and OTA-positive samples (0.5 µg/L) were used to confirm the optimal parameters. For the OTA-free sample, the fluorescent signals of the T line from seven of the different combinations were almost totally quenched by the AgNP probe. The No. 13 combination showed the maximum FI under a 0.5 µg/L OTA concentration among the seven combinations (Table S1). Therefore, the optimal parameters for the AgNP–RuNP–cLFIA sensor were 5 μL AgNP probes (OD_450_ = 1.5) and 1.2 mg/mL BSA–OTA. In addition, we also optimized the donkey antimouse IgG amounts because excess donkey antimouse IgG amounts can cause the non-appearance of the fluorescent signal on the C line. Therefore, a relatively low donkey antimouse IgG concentration of 0.2 mg/mL was suggested to be sprayed on the C line to achieve an essential fluorescence value for OTA quantitative analysis. 

The T/C ratio was used for OTA quantitative assay because it can effectively offset the effects of the inherent heterogeneity of the LFIA sensor in the present study. The accurate readout time of the T/C ratio was obtained from an immunological kinetic curve by running OTA-spiked phosphate buffer solution (PBS, 0.05 µg/L OTA). The ratio of T/C was constant after a 20 min running time, as shown in Figure S2a. We further ran the immunological kinetic curves at a 0.5 µg/L OTA concentration in grape juice and wine samples to confirm the above results. Similar results are shown in Figure S2b. Thus, the 20 min immunoreaction time was necessary to ensure the reproducibility of the quantitative analysis using cLFIA sensor.

In summary, the optimal experimental parameters of the AgNP–RuNP–cLFIA sensor were as follows: 80 μg/mL BSA-RuNP solution containing either 1.2 mg/mL BSA–OTA antigen (the mole ratio of BSA and OTA is 1:15) or 0.2 mg/mL donkey antimouse IgG was dispensed on the NC membrane as the T or C line at a spraying density of 0.75 μL/cm. A total of 70 μL of sample solution with 5.0 μL AgNP probes (OD_450_ = 1.5) was used to run the cLFIA sensor. The value of the T/C ratio of the cLFIA sensor was recorded with a commercial strip reader for OTA quantitative analysis after 20 min incubation.

### 2.4. Performance Evaluation of AgNP–RuNP–cLFIA Sensor

**Figure 4:**
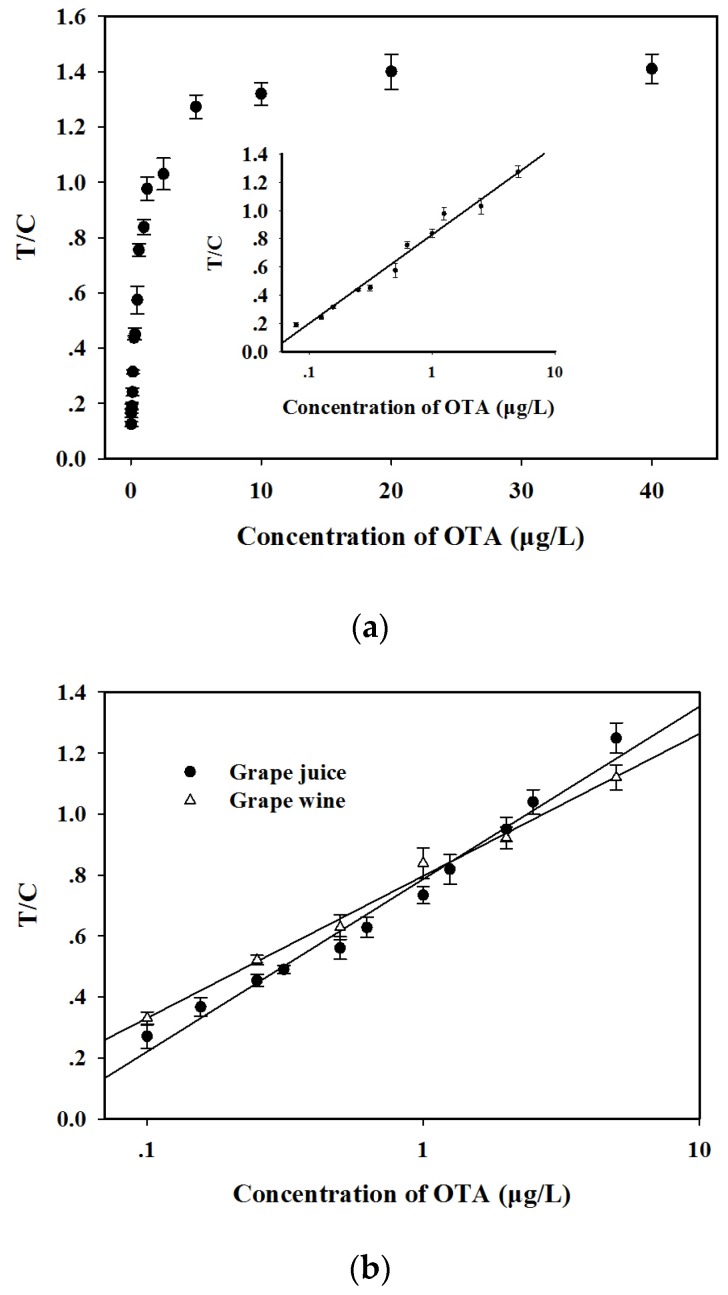
(**a**) Standard curve for OTA quantitative analysis in phosphate buffer solution (0.01 M, pH 7.0). The regression equation is: *y* = 0.272 ln*x* + 0.828 (0.08 ≤ *x* ≤ 5.0 µg/L, *R*^2^ = 0.98); (**b**) Matrix-matched standard curves for OTA quantitative analysis in red grape wine and juice samples. The regression equations are: y = 0.245 ln*x* + 0.786 (0.10 ≤ *x* ≤ 5.0 µg/L, *R*^2^ = 0.98) for grape juice sample and *y* = 0.202 ln*x* + 0.797 (0.10 ≤ *x* ≤ 5.0 µg/L, *R*^2^ = 0.99) for red grape wine sample. The vertical bars indicate the standard deviation (*n* = 3).

**Figure 5:**
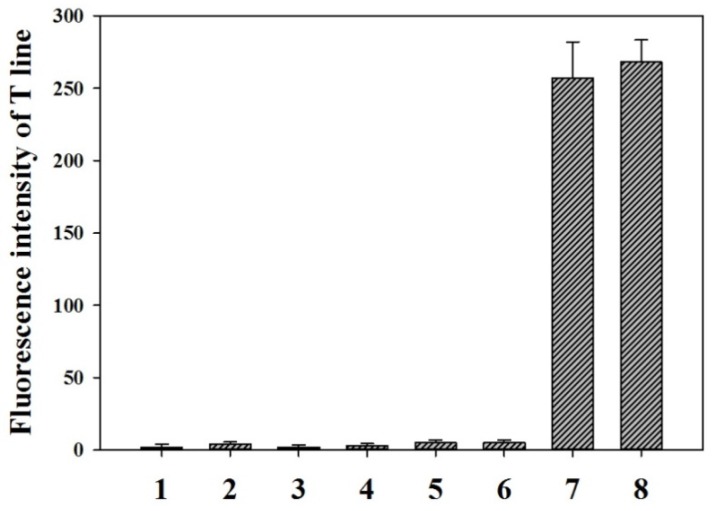
Specificity experiments for OTA, aflatoxin B_1_ (AFB_1_), patulin (PAT), citrinin (CIT), zearalenone (ZEN), deoxynivalenol (DON), and fumonisin B_1_ (FB_1_) using the AgNP–RuNP–cLFIA sensor. 1: AFB_1_ (10 µg/L), 2: PAT (10 µg/L), 3: CIT (10 µg/L), 4: ZEN (10 µg/L), 5: FB_1_ (10 µg/L), 6: DON (10 µg/L), 7: OTA (0.5 µg/L), and 8: OTA (0.5 µg/L) containing 10 µg/L of AFB_1_, PAT, CIT, ZEN, FB_1_ and DON. The vertical bars indicate the standard deviation (*n* = 3).

The calibration curve was performed by running 18 different OTA concentrations from 0 µg/L to 40 µg/L in PBS. Figure 4a shows that the T/C ratios sharply increased as the OTA concentration increased and reached a constant when the OTA concentration was more than 20 µg/L. This finding indicates that the fluorescence on the T line was completely exposed. Moreover, the data also exhibited a linear range for OTA quantitative detection between 0.08 and 5.0 µg/L. The regression equation can be represented by *y* = 0.272 ln*x* + 0.828 (0.08 ≤ *x* ≤ 5.0 µg/L, *R*^2^ = 0.98), where y is the T/C ratio and x is the logarithm of the OTA concentration. Error bars were based on three duplicate experiments. The LOD, defined as the OTA minimum concentration, for which the mean of the T/C value minus three times the standard deviation is greater than that of OTA-free sample, was calculated at 0.06 µg/L. The specificity of the proposed method was evaluated by running 0.5 µg/L OTA solution and six other common mycotoxins (10 µg/L), including aflatoxin B_1_ (AFB_1_), patulin (PAT), citrinin (CIT), zearalenone (ZEN), deoxynivalenol (DON), and fumonisin B_1_ (FB_1_). Figure 5 shows that the FI value on the T line was 268 for OTA detection. No fluorescence signals on the T line were observed for the other six mycotoxins (10 µg/L). This finding indicates that the proposed method exhibits a good specificity for OTA detection. The reliability of the AgNP–RuNP–cLFIA sensor for OTA quantitative detection was evaluated by analyzing OTA-spiked red grape wine and juice samples at three spiked levels of 0.4, 2, and 5 μg/L. The two-fold diluted juice and four-fold diluted wine samples were used to run the sensors after a simple sample pretreatment. Two matrix-matched standard curves were reconstructed to decrease the interference from the sample matrices (Figure 4b). The regression equation of grape juice was *y* = 0.245 ln*x* + 0.786 (0.10 ≤ *x* ≤ 5.0 µg/L, *R*^2^ = 0.98), whereas that of red grape wine was *y* = 0.202 ln*x* + 0.797 (0.10 ≤ *x* ≤ 5.0 µg/L, *R*^2^ = 0.99). The LODs of the AgNP–RuNP–cLFIA sensor for grape juice and wine were 0.16 μg/L (two-fold dilution) and 0.31 μg/L (four-fold dilution), respectively. The average recoveries of OTA in red grape wine ranged from 88.0% to 110.0% with a coefficient of variation (CV) ranging from 7.81% to 9.09% and those for grape juice ranged from 92.0% to 110.0% with the CV ranging from 6.82% to 8.54%, as shown in Table 1. The above results demonstrate an acceptable accuracy and precision of the AgNP–RuNP–cLFIA sensor for the quantitative analysis of OTA in real grape wine and juice samples. Although some LFIAs aimed at detecting OTA have been previously developed [2,25,26,27], to our knowledge, the proposed method is one of the most sensitive assays for effectively detecting OTA in wine and grape juice (Table S2).

## 3. Conclusions 

An AgNP-based fluorescence-quenching cLFIA sensor for the quantitative detection of OTA in grape wine and juice was successfully developed in this study. Quantitative OTA detection with the new method took only 20 min with an LOD of 0.06 µg/L in PBS. The dynamic linear range was from 0.08 µg/L to 5.0 µg/L. The proposed method also showed good accuracy and precision for the detection of low, medium, and high concentrations of OTA in real grape wine and juice samples after a simple sample pretreatment. The developed AgNP–RuNP–cLFIA sensor was well suited for on-site screening detection of OTA in grape wine and grape juice considering the speediness, low cost, and convenience.

## 4. Materials and Methods 

Materials: AgNO_3_, trisodium citrate, C_76_H_52_O_46_, PEG8000, and BSA were purchased from Sigma-Aldrich. OTA, AFB_1_, CIT, DON, ZEN, PAT, and FB_1_ were purchased from HuaanMagnech (Beijing, China). BSA–RuNPs, OTA and BSA conjugates (BSA–OTA, mole ratio of OTA and BSA is 15:1), anti-OTA monoclonal antibody (anti-OTA McAb, No. BJ02C) ascites and the filter paper were provided by Jiangxi ZodolabsBiotech. Corp. (Jiangxi, China). Donkey antimouse IgG antibodies were purchased from Zhongshan Biotechnology, Inc. (Beijing, China). The sample pad, conjugate release pad, NC membrane, and absorbent pad were obtained from Schleicher and Schuell GmbH (Dassel, Germany). All other reagents were of analytical grade or better and purchased from Sinopharm Chemical Corp. (Shanghai, China). Distilled water passed through a Millipore system (ρ = 18.2 MΩ) was used in all experiments. All glassware was first rinsed with acetone and then with Millipore water before use. 

Instruments: The BioDot XYZ platform, combined with a motion controller, BioJet Quanti3000k dispenser, and AirJet Quanti3000k dispenser for solution dispensing, was supplied by BioDot (Irvine, CA, USA). An automatic programmable cutter was purchased from Shanghai Jinbiao Biotechnology Co., Ltd. (Shanghai, China). The fluorescent strip reader was assembled by ShanghaiHuguo Science Instrument Co., Ltd. (Shanghai, China) according to our experiment requirement. Briefly, high power 450 nm LED arrays were used as excitation light source and eliminated with a 540 nm cut-off filter. The emission fluorescence of the RuNPs on the test and control lines were focused with a cylindrical lens and received by a light sensitive cell after passing through an optical slit.

Preparation of AgNPs: AgNPs were prepared according to seed growth method with some modifications [28]. Briefly, 1 mL AgNO_3_ (25 mM) was added under vigorous stirring to 100 mL of boiling aqueous solution containing 5 mM sodium citrate and 0.1 mM tannic acid. The solution immediately turned bright yellow. Up to 19.5 mL of the above solution was removed after preparation of silver seed. In addition, the remaining silver seed solution was cooled to 90 °C. Then, 19.5 mL of pure water containing 500 μL sodium citrate (25 mM), 1.5 mL tannic acid (2.5 mM), and 1 mL AgNO_3_ (25 mM) were added and maintained at 90 °C for 30 min. The AgNPs with an average diameter of 63.6 nm were obtained by repeating this process 11 times (OD_450_ = 15). The morphology and size distribution of AgNPs were characterized through high-resolution transmission electron microscopy (JEOL JEM 2100, Tokyo, Japan). The maximum SPR peaks of the AgNPs were determined using a double-beam UV-visible spectrophotometer (Cintra 10e; GBC, Braeside, Victoria, Australia).

Preparation of anti-OTA McAb-labeled AgNPs(AgNP probe): Unpurified anti-OTA ascitic fluids were used to label AgNPs directly according to the previous reports [29]. First, 5 mL of as-prepared AgNP solution was centrifuged at 7000 rpm for 10 min. The AgNP pellet was resuspended with 10 mL of 0.1 M boric acid. Then, the pH of the AgNP solution was adjusted to pH 7.5 with 0.1 M sodium hydroxide. The desired anti-OTA ascitic fluids were added dropwise to 1 mL AgNP solution to prepare the antibody-labeled AgNP probe. The AgNP probe was centrifuged at 7000 rpm for 10 min after incubation at room temperature for 2 h on a rolling mixer. The pellet was resuspended with 1 mL of 0.1 M borate buffer containing 0.1% *w*/*v* BSA (pH 7.5). The above solution was centrifuged at 7000 rpm for 10 min after 5 min incubation. The as-prepared AgNP probe was also resuspended with 0.1 M borate buffer to OD_450_ value at 1.5 for further use.

Preparation of fluorescence-quenching AgNP–RuNP–cLFIA sensor: The fluorescence-quenching AgNP–RuNP–cLFIA sensor was prepared according to a previous report with some modifications. The cLFIA sensor comprised five parts, including NC membrane, sample pad, conjugate release pad, filter paper and absorbent pad as shown in Figure 1a. The sample pads were saturated with 20 mmol/L sodium borate buffer (pH 8.0) containing 1.0% (*w*/*v*) BSA, 0.25% Tween-20, and 0.1% (*w*/*v*) NaN_3_ and then dried overnight at room temperature with controlled relative humidity lower than 45% through a dehumidifier. Meanwhile, a filter paper was added into the pretreated sample pad to filter the impurities in the grape juice and wine. The conjugate release pad was pretreated with 0.01 M PBS (pH 7.2) containing 0.25% Tween-20 and 4% sucrose and then dried at 60 °C for 2 h. One BSA–RuNP solution (80 μg/mL) containing 1.2 mg/mL BSA–OTA and another BSA–RuNP solution (80 μg/mL) containing 0.2 mg/mL donkey antimouse IgG solutions were sprayed on the NC membrane as the T and C lines. The dispensing density was 0.75 μL/cm. The distance between the T and C lines was 5 mm. The NC membrane was dried at 37 °C for 12 h. Then, the NC membrane, conjugate release pad, sample pad, filter paper, and absorbent pad were laminated and pasted into a plastic backing plate and then cut into 4 mm-wide strips. Each strip was inserted into rigid plastic cassettes with a sample well and a reading window and then sealed in a plastic bag with desiccant gel for further use.

Immunological kinetics analysis of the AgNP–RuNP–cLFIA sensor: The development of FIs on the T and C lines can indirectly reflect the immunological dynamic interaction between AgNP probe and BSA–OTA on the T line as well as those of AgNP probe and donkey antimouse IgG on C lines. The kinetic curve was determined according to our previous report. Briefly, approximately 5.0 μL AgNP probe and 70 μL sample solution containing 0.05 µg/L of OTA were premixed at room temperature for 3 min and then pipetted into the strip sample well. The strip was inserted into a fluorescent strip reader after 2 min incubation. The FIs of the T, C, and T/C ratio were recorded every 30 s for 30 min. The kinetic curves were established by plotting the development of FI_T_, FI_C_, and the T/C ratio against immunoreaction time.

Quantitative detection of OTA using AgNP–RuNP–cLFIA sensor: OTA quantitative analysis was performed by adding the mixture of 5.0 μL AgNP probe and 70 μL sample solution into the well of the sensor. The cLFIA sensor was scanned with a commercial strip reader after 20 min incubation. The T/C ratio values were also recorded. The calibration curves of OTA in grape juice and wine were determined by running twelve standard OTA solutions. The OTA standard solutions were prepared by spiking OTA stock solution to OTA-free solutions of grape juice and wine. The final OTA concentration in the pretreated diluting solution was 0, 0.1, 0.15, 0.25, 0.3, 0.5, 0.6, 1.0, 1.25, 2.0, 2.5, and 5.0 µg/L. The matrix-matched standard curves were constructed by plotting the T/C ratios against the logarithm of OTA concentrations. The OTA concentration in grape juice or wine was calculated according to the different matrix-matched standard curves.

Sample preparation: OTA-free grape juice and wine samples were obtained from the local Metro mall and Wal-Mart (Jiangxi, China). The sample was pretreated according to a previous report with slight modification [25]. Briefly, the desired OTA concentration was added to grape juice and wine samples. The spiked grape juices were diluted twice with 40 mM NaHCO_3_ solution containing 0.5% polyethylene glycol (PEG). The wines were diluted four times with 40 mM NaHCO_3_ solution containing 1% PEG.

## Figures and Tables

**Table 1:** Precision and accuracy of the AgNP–RuNP–cLFIA sensor for OTA spiked blank red grape wine and juice samples.

Sample	Spiking Levels (μg/L)	Dilution Factors	Mean Value (μg/L) *	SD %	CV %	Recovery %
Grape wine	0.40	4	0.11	0.94	8.54	110.0
	2.00	4	0.44	3.01	6.84	88.0
	5.00	4	1.26	2.28	1.81	100.8
Grape juice	0.40	2	0.22	0.76	3.45	110.0
	2.00	2	0.92	3.59	3.90	92.0
	5.00	2	2.56	4.80	1.87	102.4

* Mean value of three replicates.

## Supplementary Materials

The following are available online at www.mdpi.com/2072-6651/9/3/83/s1, Table S1: Optimization of AgNP probe contents and BSA–OTA concentrations, Table S2: Comparison of the published LFIA with AgNP–RuNP–cLFIA sensor for OTA detection, Figure S1: Fluorescence intensity values of test and control line zones under a series of RuNP–bovine serum albumin concentration recorded with a commercial strip reader (underneath) and the corresponding stereogram of the lateral flow immunoassay sensor under a 450 nm LED light excitation, Figure S2: (a) Immunoreaction kinetic curves of FIT, FIC, and T/C ratio by running in phosphate buffer solution containing 0.05 ng/mL of OTA; (b) Immunoreaction kinetic curves of T/C ratio by running red grape wine and juice samples containing 0.5 ng/mL of OTA.
