# Supplementary material for: Silver Nanoparticle-Based Fluorescence-Quenching Lateral Flow Immunoassay for Sensitive Detection of Ochratoxin A in Grape Juice and Wine
Source: Toxins (Basel). 2017 Feb 28;9(3):83. doi: 10.3390/toxins9030083 (PMC5371838; doi:10.3390/toxins9030083)
Supplement: Supplementary file 1 [file toxins-09-00083-s001.pdf]

Supplementary Materials: Silver Nanoparticle-based Fluorescence-quenching Lateral Flow Immunoassay for Sensitive Detection of Ochratoxin A in Grape Juice and Wine

Hu Jiang, Xiangmin Li, Ying Xiong, Ke Pei, Lijuan Nie and Yonghua Xiong

Table S1. Optimization of AgNP probe contents and BSA–OTA concentrations

No.	Volume of AgNPprobes (μL) [a]	OTA-BSA Con. (mg/mL)	FI_T (OTA-free sample)	FI_T (0.5 ng/mL of OTA sample)
1	3	0.4	340	398
2	3	0.8	226	326
3	3	1.2	264	341
4	3	1.6	211	336
5	3	2.0	187	259
6	4	0.4	269	403
7	4	0.8	134	274
8	4	1.2	216	302
9	4	1.6	202	259
10	4	2.0	149	192
11	5	0.4	197	331
12	5	0.8	62.4	254
13 [b]	5	1.2	0.00	254
14	5	1.6	0.00	216
15	5	2.0	0.00	173
16	7.5	0.4	149	269
17	7.5	0.8	0.00	120
18	7.5	1.2	0.00	173
19	7.5	1.6	0.00	139
20	7.5	2.0	0.00	0.00

a: OD_{450} value of AgNP probes is 1.5; b: the optimal parameter combination.

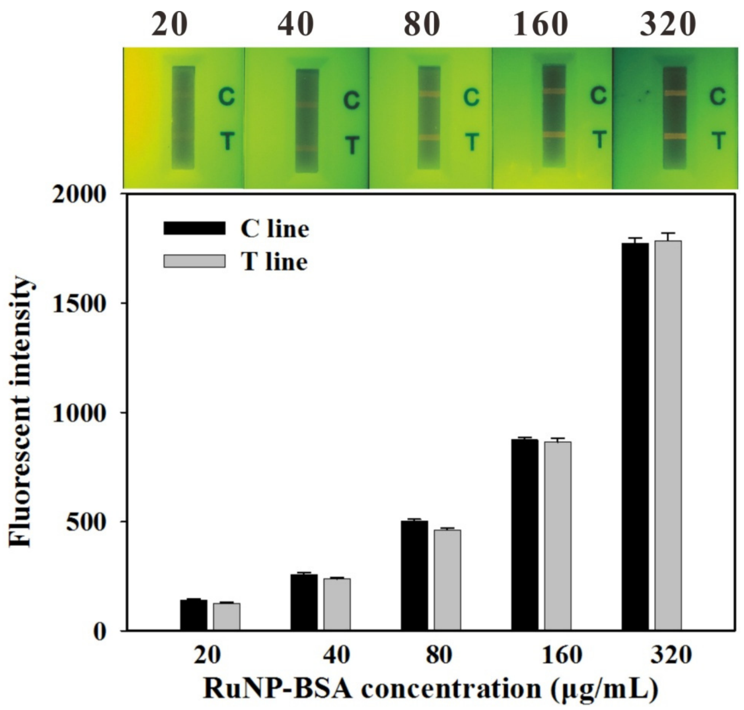

Figure S1. Fluorescence intensity values of test and control line zones under a series of RuNP–bovine serum albumin concentration recorded with a commercial strip reader (underneath) and the corresponding stereogram of the lateral flow immunoassay sensor under a 450 nm LED light excitation (above). The vertical bars indicate the standard deviation (n = 3).

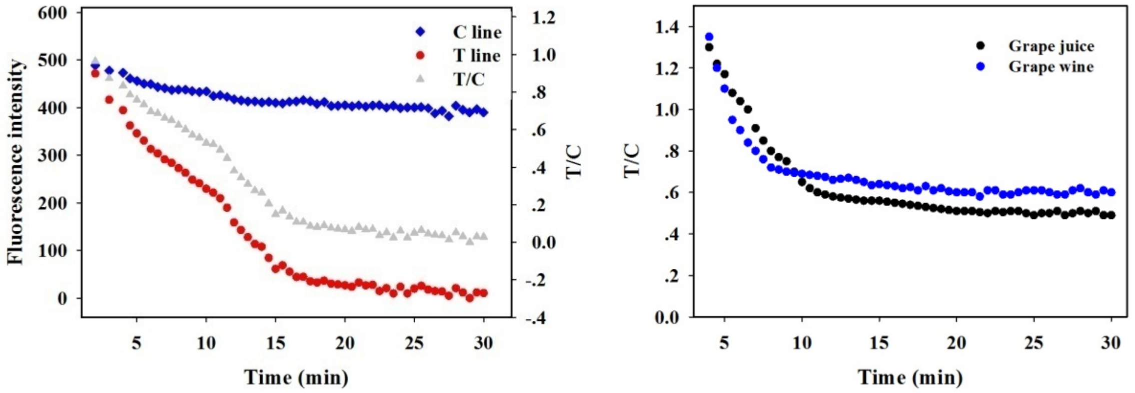

(**a**) (**b**)

Figure S2. (**a**) Immunoreaction kinetic curves of FI_T, FI_C, and T/C ratio by running in phosphate buffer solution containing 0.05 ng/mL of OTA; (**b**) Immunoreaction kinetic curves of T/C ratio by running red grape wine and juice samples containing 0.5 ng/mL of OTA.

Table S2. Comparison of the published LFIA with AgNP–RuNP–cLFIA sensor for OTA detection.

Reference	Label/ signal	LOD (μg/kg)	Samples	LFIA type
Lai et al.[1] (2009)	AuNPs	10	Cereal	Qualitative
Anfossi et al. [2] (2011)	AuNPs	1.5	Cereal	Qualitative
Anfossi et al.[3] (2012)	AuNPs	1<br>1	Grape juice<br>wine	Qualitative
Sun et al. [4] (2016)	AuNPs	0.77	Cereal	Qualitative
This study	AgNPs/RuNPs	0.16<br>0.31	Grape juice<br>wine	Qualitative

References

1. Lai, W.; Fung, D.Y.C.; Yang, X.; Renrong, L.; Xiong, Y. Development of a colloidal gold strip for rapid detection of ochratoxin A with mimotope peptide. *Food Control* **2009**, *20*, 791–795.
2. Anfossi, L.; D'Arco, G.; Baggiani, C.; Giovannoli, C.; Giraudi, G. A lateral flow immunoassay for measuring ochratoxin A: development of a single system for maize, wheat and durum wheat. *Food Control* **2011**, *22*, 1965–1970.
3. Anfossi, L.; Giovannoli, C.; Giraudi, G.; Biagioli, F.; Passini, C.; Baggiani, C. A Lateral flow immunoassay for the rapid detection of ochratoxin A in wine and grape must. *J. Agr. Food Chem.* **2012**, *60*, 11491–11497.
4. Sun, Y.; Xing, G.; Yang, J.; Wang, F.; Deng, R.; Zhang, G.; Hu, X.; Zhang, Y. Development of an immunochromatographic test strip for simultaneous qualitative and quantitative detection of ochratoxin A and zearalenone in cereal. *J. Sci. Food Agric.* **2016**, *96*, 3673–3678.
